# Pancreatic tumours: molecular pathways implicated in ductal cancer are involved in ampullary but not in exocrine nonductal or endocrine tumorigenesis

**DOI:** 10.1054/bjoc.2000.1567

**Published:** 2001-01

**Authors:** P S Moore, S Orlandini, G Zamboni, P Capelli, G Rigaud, M Falconi, C Bassi, N R Lemoine, A Scarpa

**Affiliations:** Department of Pathology, Department of Surgery, Università di Verona, Italy; Imperial Cancer Research Fund Molecular Oncology Unit, Imperial College School of Medicine, London, UK

**Keywords:** pancreas, carcinoma, intraductal papillary-mucinous tumour, acinar cancer, solid pseudopapillary tumour, ampulla of Vater cancer, endocrine tumour, K-*ras*, *p16*, *p53*, *DPC4/Smad4*, microsatellites, allelotyping

## Abstract

Alterations of K-*ras*, *p53*, *p16* and *DPC4/Smad4* characterize pancreatic ductal cancer (PDC). Reports of inactivation of these latter two genes in pancreatic endocrine tumours (PET) suggest that common molecular pathways are involved in the tumorigenesis of pancreatic exocrine and endocrine epithelia. We characterized 112 primary pancreatic tumours for alterations in *p16* and *DPC4* and immunohistochemical expression of DPC4. The cases included 34 PDC, 10 intraductal papillary-mucinous tumours (IPMT), 6 acinar carcinomas (PAC), 5 solid-pseudopapillary tumours (SPT), 16 ampulla of Vater cancers (AVC) and 41 PET. All tumours were also presently or previously analysed for K- *ras* and *p53* mutations and allelic loss at 9p, 17p and 18q. Alterations in K- *ras*, *p53*, *p16* and *DPC4* were found in 82%, 53%, 38% and 9% of PDC, respectively and in 47%, 60%, 25% and 6% of AVC. Alterations in these genes were virtually absent in PET, PAC or SPT, while in IPMT only K-*ras* mutations were present (30%). Positive immunostaining confirmed the absence of *DPC4* alterations in all IPMT, SPT, PAC and PET, while 47% of PDC and 38% of AVC were immunonegative. These data suggest that pancreatic exocrine and endocrine tumourigenesis involves different genetic targets and that among exocrine pancreatic neoplasms, only ductal and ampullary cancers share common molecular events. © 2001 Cancer Research Campaign http://www.bjcancer.com


*British Journal of Cancer* (2001) **84**(2), 253–262
© 2001 Cancer Research Campaign
doi: 10.1054/ bjoc.2000.1567, available online at http://www.idealibrary.com on IDEAL®

### Pancreatic tumours: molecular pathways implicated in ductal cancer are involved in ampullary but not in exocrine nonductal or endocrine tumorigenesis

**PS Moore[1], S Orlandini[1], G Zamboni[1], P Capelli[1], G Rigaud[1], M Falconi[2], C Bassi[2], NR Lemoine[3] and A Scarpa[1]**

[1]Department of Pathology and [2]Department of Surgery, Università di Verona, Italy; [3]Imperial Cancer Research Fund Molecular Oncology Unit, Imperial College School of Medicine, London, UK

**Summary** Alterations of K-*ras*, *p53*, *p16* and *DPC4/Smad4* characterize pancreatic ductal cancer (PDC). Reports of inactivation of these latter two genes in pancreatic endocrine tumours (PET) suggest that common molecular pathways are involved in the tumorigenesis of pancreatic exocrine and endocrine epithelia. We characterized 112 primary pancreatic tumours for alterations in *p16* and *DPC4* and immunohistochemical expression of DPC4. The cases included 34 PDC, 10 intraductal papillary-mucinous tumours (IPMT), 6 acinar carcinomas (PAC), 5 solid-pseudopapillary tumours (SPT), 16 ampulla of Vater cancers (AVC) and 41 PET. All tumours were also presently or previously analysed for K-*ras* and *p53* mutations and allelic loss at 9p, 17p and 18q. Alterations in K-*ras*, *p53*, *p16* and *DPC4* were found in 82%, 53%, 38% and 9% of PDC, respectively and in 47%, 60%, 25% and 6% of AVC. Alterations in these genes were virtually absent in PET, PAC or SPT, while in IPMT only K-*ras* mutations were present (30%). Positive immunostaining confirmed the absence of *DPC4* alterations in all IPMT, SPT, PAC and PET, while 47% of PDC and 38% of AVC were immunonegative. These data suggest that pancreatic exocrine and endocrine tumourigenesis involves different genetic targets and that among exocrine pancreatic neoplasms, only ductal and ampullary cancers share common molecular events. © 2001 Cancer Research Campaign http://www.bjcancer.com

**Keywords**: pancreas; carcinoma; intraductal papillary-mucinous tumour; acinar cancer; solid pseudopapillary tumour; ampulla of Vater cancer; endocrine tumour; K-*ras*; *p16*; *p53*; *DPC4/Smad4*; microsatellites; allelotyping

Common pancreatic ductal adenocarcinoma (PDC) is characterized by a relatively unique molecular fingerprint constituted by frequent alterations of the K-*ras* (Almoguera et al, 1988; Lemoine et al, 1992;), *p53* (Barton et al, 1991; Kalthoff et al, 1993; Scarpa et al, 1993a), *p16*$^{INK4a}$ (Caldas et al, 1994) and *DPC4/Smad4* genes (Hahn et al, 1996). Numerous studies on K-*ras* and *p53* have confirmed that they are mutated at high frequency in primary PDC (K-*ras* reviewed in Hruban et al, 1993 and Scarpa et al, 1994a; for *p53* see: Pellegata et al, 1994; Redston et al, 1994). Less information is available regarding alterations of *p16* and *DPC4* in primary PDC and the only two available reports suggested that alterations in these two genes occur less frequently in primary PDC than in derived cell lines (Huang et al, 1996; Bartsch et al, 1999a). Studies on xenografts and cell lines have shown that homozygous deletions may account for up to half of all instances of inactivation of these genes (Caldas et al, 1994; Hahn et al, 1996; Naumann et al, 1996; Villanueva et al, 1998) although this phenomenon is difficult to demonstrate in primary PDC due to the high admixture of nonneoplastic cells in these tissues. In addition, the *p16* gene may also be silenced by promoter methylation (Schutte et al, 1997), an epigenetic event not yet examined in a series of primary PDC.

Very little is known about the molecular abnormalities in neoplasms other than ductal arising from the exocrine pancreas, including intraductal papillary-mucinous tumours (IPMT), solid-pseudopapillary tumours (SPT), the cystic group of malignancies, the extremely rare acinar cell carcinomas (PAC), and cancers arising from the ampulla (papilla) of Vater (AVC) (Klöppel et al, 1996; Solcia et al, 1997).

Based on the mutational analysis of the K-*ras* and *p53* genes in 35 PDC, 6 pancreatic exocrine nonductal and 12 pancreatic endocrine tumours (PET), an earlier study concluded that the molecular pathogenesis of exocrine nonductal and endocrine tumours involves pathways different from those involved in PDC (Pellegata et al, 1994). However, two recent reports of highly frequent alterations of the *p16* and *DPC4* genes in PET suggested that common molecular pathways are involved in the tumorigenesis of exocrine and endocrine epithelia (Muscarella et al, 1998; Bartsch et al, 1999b).

To address these issues specifically, we have analysed 112 primary pancreatic tumours of different types, including 34 PDC, 37 exocrine nonductal and 41 PET, for molecular alterations in *p16* and *DPC4* genes. The inactivation of the *DPC4* gene by additional mechanisms such as homozygous deletion was studied using immunohistochemistry, which has been shown to correlate with inactivation of the gene (Wilentz et al, 2000). All tumours were also presently or previously analysed for K-*ras* and *p53* mutations and allelic loss at chromosomal arms 9p, 17p and 18q at sites linked to *p16*, *p53* and *DPC4* genes. This study represents the largest report to date on the mutational status of different pancreatic tumour types for the 4 genes most commonly altered in pancreatic ductal cancers and provides evidence that only ductal and ampulla of Vater cancers share common molecular anomalies, while the less common tumour entities have a distinct molecular pathogenesis.

*Received 3 May 2000*
*Revised 28 September 2000*
*Accepted 28 September 2000*

*Correspondence to:* A Scarpa

### MATERIALS AND METHODS

All the studies performed were approved by the Ethics Committee of Verona University.

### Primary tumours

The 112 frozen pancreatic tumours consisted of 34 PDC, 37 exocrine nonductal and 41 PET. The nonductal exocrine tumours were composed of 10 1PMT, 6 PAC, 5 SPT and 16 AVC. The PETs included 30 nonfunctional and 11 functional tumours. A neoplastic cellularity of at least 60% for PDC and ranging from 70% to virtually 100% for all other tumour types was obtained in all cases by either cryostat enrichment or microdissection of the frozen tumour samples, as described (Achille et al, 1996a; Sorio et al, 1999).

Among the 34 PDC, 18 cases had been previously analysed for mutations in the K-*ras* and *p53* genes; in particular, PDC3-PDC6 and PDC9-PDC11 were described in Achille et al (Achille et al, 1996a) and PDC19-PDC29 correspond to previously reported cases 1, 4, 5, 7, 8, 10, 11, 12, 14, 15 and 21 (Scarpa et al, 1993a). The remaining 16 cases have not been previously reported.

The 10 IPMT were characterized by diffuse or segmental dilatation of the main and/or branch pancreatic ducts with intraductal growth pattern sometimes forming intraluminal masses, which presented a wide spectrum of modifications ranging from low-to high-grade dysplasia and from carcinoma in situ to invasive cancer (Klöppel et al, 1996; Solcia et al, 1997). In particular, 3 cases had only the intraductal component and were considered of borderline malignancy, according to established criteria (Klöppel et al, 1996; Solcia et al, 1997), 7 cases also had an invasive component which was represented by muconodular carcinoma in 6 and ductal-like cancer in one. Cases IPMT1-IPMT4 correspond to cases 4, 5, 2 and 3, respectively, of a previous report in which they had been analysed for K-*ras* and *p53* mutations (Sessa et al, 1994).

The 5 SPT were from prepubertal girls or young women, and characteristically showed progesterone receptor immunostaining (Zamboni et al, 1993). These cases have not been previously reported.

The 6 PAC were diagnosed by histopathological criteria and cell marker analysis. The latter confirmed the acinar nature of the neoplastic cells, which expressed lipase in all cases, amylase in 2 cases, trypsin in 5 and chymotrypsin in 4 cases (Klimstra et al, 1992; Hoorens et al, 1993; Solcia et al, 1997). These cases have not been previously reported.

The 16 AVC were selected by applying strict topographical criteria obtained at gross and histological examinations. Only small lesions with unequivocal ampullary origin, topographically centred in the region of the papilla of Vater were included in the study (Achille et al, 1996b, 1998; Scarpa et al, 1993b, 1994b). AVC showing microsatellite instability of the type seen in replication error phenotype (RER+) cancers were excluded from the study (Achille et al, 1997). All cases except AVC16 have been previously described and analysed for mutations in the K-*ras* and *p53* genes and for 17p and 18q LOH (Scarpa et al, 2000).

The series of 41 PET was composed of 30 nonfunctional and 11 functional tumours, including 9 insulinomas, 1 gastrinoma and 1 VIPoma. 23 tumours were benign and 18 malignant, in accordance with the respective absence or presence of invasion of the neighbouring organs and/or nodal/distant metastases, as evaluated by imaging techniques, surgical and pathological examinations. All tumours were characterized using a panel of monoclonal antibodies recognizing pan-endocrine markers (chromogranin A, synaptophysin and non-specific enolase) and gastrointestinal hormones (insulin, glucagon, somatostatin, pancreatic polypeptide, gastrin, serotonin, and vasoactive intestinal peptide). Only those tumours giving rise to an endocrine syndrome were considered as functional. PETs were considered nonfunctional if clinical symptoms were absent, regardless of the immunostaining results. Among the 30 nonfunctional tumours, 24 showed no immunoreactivity for the tested hormones, while 4 tested positive for glucagon and 2 for somatostatin. Mutations in the K-*ras* and *p53* genes for tumours NF1–NF10 and F1–F6 have been previously reported (Beghelli et al, 1998). All other cases have not been previously reported.

### Loss of heterozygosity analysis

Most cases were analysed for allelic loss with high-molecular weight DNA, although some analyses were performed with DNA extracted from paraffin-embedded tissues prepared as described (Scarpa et al, 2000). Two microsatellites each were used for chromosomal arms 9p, 17p, and 18q. All cases were analysed for allelic loss using the microsatellite markers *D9S171*, *D9S161*, *D17S799*, and *D18S474*. For high molecular weight DNA, the markers *D17S938* and *D18S64* were also used. For paraffin-embedded samples, the markers *D17S1857* and *D18S1102* were used as alternatives. These markers are part of the AB1 Prism, Linkage Mapping Set, ver. 1 and 2 (Perkin Elmer). PCR products were pooled and electrophoresed on an AB1 Prism 377 instrument. Electropherograms were analysed for microsatellite alterations using GeneScan v. 3.1 and Genotyper v. 2.5 software (Perkin Elmer). Only microsatellites showing two distinct alleles in normal DNA were considered as informative. LOH was scored when there was loss of intensity of one allele in the tumour sample with respect to the matched allele from normal tissue and the relative intensity of the 2 alleles in the tumour DNA differed from the relative intensity in the non-neoplastic tissue DNA by a factor of at least two. All the analyses were verified by visual examination. Microsatellites showing differently sized alleles compared with their respective normal sample were scored as unstable. LOH on each chromosomal arm was scored in cases showing allelic loss in at least one informative marker for that arm.

### Mutational analysis of K-*ras*, *p16*, and *p53* and *DPC4*

All samples were analysed for mutations in exon 1 of the K-*ras* gene, exons 1 and 2 of *p16*, exons 5–9 of *p53*, and exons 8–11 of *DPC4* by single-strand conformation polymorphism (SSCP) and direct sequencing of PCR amplified DNA fragments. PCR was performed in a volume of 10 µl using 40 ng DNA with 35 cycles. Primers for amplification of the *p53* (Scarpa et al, 1993a), *p16* (Zhang et al, 1994), K-*ras* (Scarpa et al, 1994b) and *DPC4* genes (Hahn et al, 1998) were as described. For SSCP analysis, 0.1 µl [$\alpha$-$^{32}$P]-dCTP (3000 Ci mmol$^{-1}$) was added to the amplifications. Samples were run on 5% polyacrylamide gels containing 0.125% bis-acrylamide with and without 5% glycerol at 30 W. Bands exhibiting aberrant migration were cut from the gel, re-amplified, and sequenced on an AB1 Prism 377 instrument. The DNA from 6 xenografted control samples were a kind gift of Dr S Hahn, University of Bochum, Germany (Hahn et al, 1996).

© 2001 Cancer Research Campaign

### Additional analysis for *DPC4* mutations

As we found a relatively low number of *DPC4* mutations using the PCR-amplified fragments described (Hahn et al, 1998), a second round of PCR-SSCP for exons 8–11 was carried out using smaller amplified fragments to verify the sensitivity of SSCP. Exons 8–11 were each divided into two fragments, each less than 180 bp. The primers used and the size of the amplified fragments were as follows. Exon 8A (167 bp): DPC4Ex8B, AAGCCT-TATATCTTTCTCATGG, DPC4AS18, GAAGGGTCCACG-TATCCAT; Exon 8B (157 bp): DPC4S18, GTTCCTTC-AAGCTGCCCTAT, DPC4AS8, CAATTTTTTAAAGTAACTATC TGA; Exon 9A (175 bp): DPC4Ex9B, CTATACAATCTGAAG-TAAAATT, DPC4AS19, AGTAGTAACTCTGTACAAAG; Exon 9B, (173 bp): DPC4S19, TTGGGTCAGGTGCCTTAGT, DPC4Ex9, TTTTGACAACAAATAGAGCTTTAAGTC; Exon 10A (145 bp): DPC4Ex10, GAATTTTCTTTATGAACTCATAG, DPC4IAS10, GGATGTTTCCTGCCACGGC; Exon 10B (155 bp): DPC41S10, CACAAGCTGCAGCAGCTGCC, DPC4-Ex10B, ATCAACTGAGTAAATCAAGATAA; Exon 11A (179 bp): DPC4S11B, TCACCCTGTCCCTCTGATG, DPC41AS11, AGTGAATTTCAATCCAGCAA; Exon 11B (176 bp): DPC-41S11, TTACCCAAGACAGAGCATCA, DPC4Ex11, TATTTT-GTAGTCCACCATC.

### Methylation analysis of *p16*

Methylation-specific PCR for the 5' CpG island of the *p16* gene was carried out as described (Herman et al, 1996). DNA from the cell-line PaTul (kindly provided by Dr M von Bulow, University of Mainz, Germany) was used as a positive control and for evaluation of the sensitivity of methylation-specific PCR under the conditions used.

### Immunohistochemistry for p16 and DPC4

Immunohistochemistry for p16 was performed using 3 different mouse monoclonal antibodies (p16 Ab G175-405 from BD PharMingen, San Diego, USA; Neomarker p16 Ab-4 (clone 16P04) from Lab Vision Corporation, Fremont, USA; Santa Cruz p16 (F-12) from Autogen Bioclear, Calne, UK) at dilutions from 1:10 to 1:100 using different antigen retrieval and signal enhancing procedures. Immunohistochemistry for DPC4/Smad4 was performed using a mouse monoclonal antibody (Santa Cruz Smad4 (B-8) from Autogen Bioclear, Calne, UK) at a dilution of 1:100 following antigen retrieval by microwaving for 3 times 10 min each in 10 mM citrate buffer, pH 6.

The immunolabelling for DPC-4 was scored as positive or negative. Tumours scored as positive showed diffuse cytoplasmic staining of the tumour epithelium and had scattered positive nuclei. Tumours scored as negative showed no detectable cytoplasmic or nuclear DPC-4 protein. Normal pancreatic structures in the same sections served as positive controls. Cases with focal loss of expression were scored as focal positive.

### RESULTS

All 112 primary tumours were analysed for mutations in the *DPC4* and *p16* genes. Representative SSCP analyses are shown in Figures 1 and 2. The *p16* gene was also examined for methylation of its 5' CpG island by methylation-specific PCR, a representative example of which is shown in Figure 3. Under the conditions used, *p16* methylated sequences could be detected when they represented 2% or greater of the total input DNA (data not shown). All 112 tumours were presently or previously analysed for mutations in K-*ras* and *p53* genes. The 53 previously reported cases included 18 PDC, 4 IPMT, 15 AVC and 16 PET (details in Materials and Methods). All tumours were also examined for allelic loss on chromosomal arms 9p, 17p and 18q at sites linked to *p16*, *p53* and *DPC4*, respectively. Representative examples of this analysis are shown in Figure 4.

The status of *p16* and *DPC4* genes was also analysed by immunohistochemistry. However, none of the 3 anti-p16 antibodies consistently stained any structure or cell in normal pancreas with the exception of the Ab-4 from Neomarkers, which only showed positivity in variable portions of the islets of Langerhans (data not shown) confirming previous observations (Nielsen et al, 1999). Thus, the analysis of *p16* status by immunohistochemistry was not feasible, as any observed negativity in neoplastic cells could not be interpreted in the absence of reliable positive controls (see also: Wilentz et al, 1998; Geradts et al, 2000). On the other hand, the anti-DPC4 antibodies efficiently immunostained all normal pancreatic acinar, ductal and islet cells, which showed strong to intermediate cytoplasmic reactivity with occasional nuclear labelling. Representative examples of immunohistochemistry with anti-DPC4 antibodies are shown in Figure 5.

### Ductal carcinomas

The results of the analysis of 34 cases are summarized in Table 1. Mutations in K-*ras* and *p53* were found in 82% and 53% of cases, respectively. Either methylation or mutation of the *p16* gene was observed in 38% of cases. All mutations were somatic in nature with the exception of the *p16* gene alteration in case PDC16, which was germline in origin (Moore et al, 2000b). Mutations in *DPC4* were found in 9% of cases. Two recently identified polymorphisms in the *DPC4* gene were also found (Table 1) (Moore et al, 2000a). To rule out the possibility that we were not able to detect sequence variants with the PCR fragments used for SSCP, each exon was subsequently analysed using two fragments less than 180 bp in length and direct DNA sequencing was performed. No additional mutations were found. As a further control, we were able to detect all *DPC4* mutations by our PCR-SSCP conditions in 6 xenografted PDC (data not shown) kindly furnished by Dr S Hahn (Hahn et al, 1996). Allelic loss on chromosomal arms 9p, 17p, and 18q was found in 67%, 77%, and 65% of cases, respectively (Table 1). Immunohistochemical staining for DPC4 revealed that 14 cases (47%) were negative for the protein, including one case showing focal positivity. All the remaining 16 cases tested had diffusely positive labelling (Fig. 5).

### Intraductal-papillary-mucinous, solid-pseudopapillary and acinar tumours

The data regarding these exocrine nonductal tumours are summarized in Table 2. In the 10 IPMT, 3 cases were found to have K-*ras* mutation at codon 12 with no mutations in *p16*, *p53* or *DPC4*. No mutations in any of the 4 genes were found in PAC or SPT. All the 5 PAC tested showed strong to intermediate cytoplasmic immunostaining with anti-DPC4 antibodies (Fig. 5). Both invasive and all noninvasive components of the tested IPMT labelled positively for DPC4, as was seen in the 4 tested cases of SPT.

*© 2001 Cancer Research Campaign*

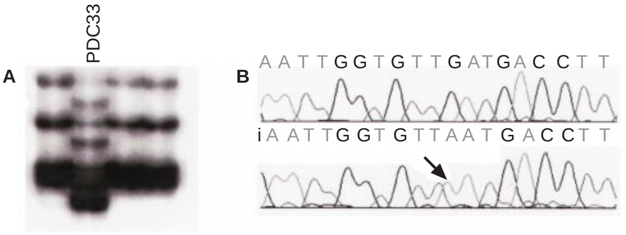

**Figure 1** Example of PCR-SSCP analysis of *DPC4*. **A**, SSCP analysis. Only the case showing aberrant band migration is indicated. The tumour showing altered migration is a ductal cancer. **B**, sequence analysis of a band not showing altered migration (top panel) and of the band displaying aberrant migration. The sequence substitution is indicated by an arrow. The nucleotide change, c1477G>A, results in the substitution of asp to asn at codon 493

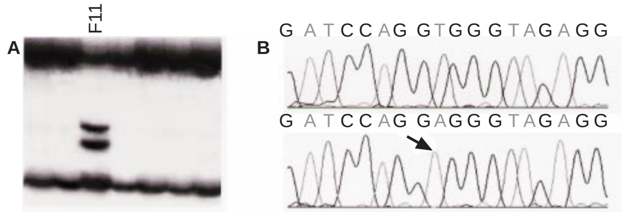

**Figure 2** Example of PCR-SSCP analysis of *p16* in primary tumours. **A**, SSCP analysis. Only the case showing aberrant band migration is indicated. The tumour showing altered migration is an insulinoma (PET-F11). **B**, sequence analysis of a band not showing altered migration (top panel) and of the band displaying aberrant migration. The sequence substitution is indicated by an arrow. The T>A substitution (g152) results in a splicing error

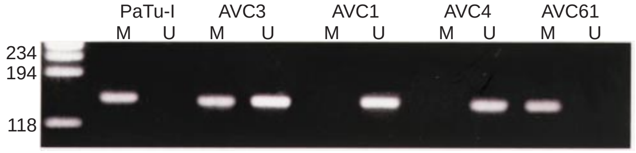

**Figure 3** Representative examples of methylation-specific PCR of the *p16* gene. In panels **A** and **B** case numbers are as indicated. M, PCR with primers specific for methylated DNA. U, PCR with primers specific for unmethylated DNA. The numbers indicate the size of the DNA marker in base pairs. The expected sizes of the PCR products are 150 bp for *p16* unmethylated DNA and 151 bp for methylated sequences. Methylation-specific PCR was carried out as described (Herman et al, 1996)

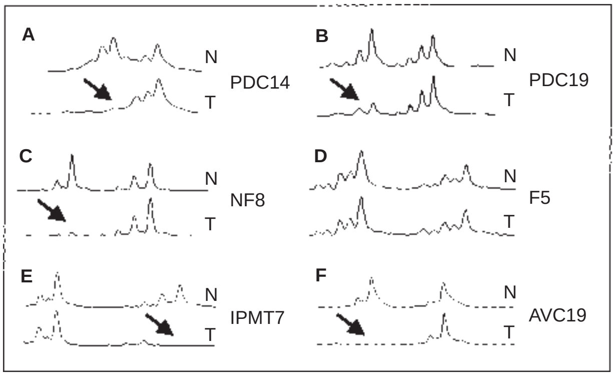

**Figure 4** Representative examples of microsatellite analysis of microdissected cancers. Case numbers are as indicated to the right of each pair of electropherogram tracings. NF, nonfunctional PET, F, functional PET. N, normal; T, tumour. All samples show allelic loss (indicated by arrows) except for the case in D. In B, it can be noticed that a neoplastic cellularity of 100% has not been achieved. The microsatellites used are as follows. A, B: *D9S171*. C, D: *D17S938*. E, F: *D18S474*

### Ampulla of Vater cancers

The results of the mutational analysis in the 16 AVC are summarized in Table 3. Mutations of K-*ras* and *p53* were found in 47% and 60% of cases, respectively. One case had a mutation of the *DPC4* gene (6%), while 4 cases (25%) had inactivation of *p16*, all of which were due to de novo methylation (Fig. 3). Immunohistochemical staining for DPC4 revealed that 6 cases (38%) were negative, including one case showing focal positivity. The remaining 10 cases were all diffusely positive. Allelic loss on chromosomal arms 9p, 17p, and 18q was detected in 13%, 63% and 50% of cases. Our genetic and immunohistochemical analysis showed that nine of 16 AVC had alterations in at least 2 of the 4 genes analysed, and that all cases showing either *p16* or *DPC4* alteration always showed alteration of either K-*ras* or *p53*.

### Endocrine tumours

The data on the endocrine tumours is shown in Table 4. One nonfunctioning (NF) case had a mutation in K-*ras* and another NF tumour had a mutated *p53* gene (Beghelli et al, 1998). One insulinoma was found to harbour a *p16* mutation affecting a splice junction (Fig. 2). 4 patients (10%) had the A148T polymorphism in the *p16* gene. No alterations were found in *DPC4*. All of the 36 cases tested stained positively for DPC4 antigen.

### DISCUSSION

The results of the present extensive molecular analysis of different primary pancreatic tumour types may be summarized as follows: a) a relatively high frequency of DNA sequence alterations of K-*ras*, *p53* and *p16* was detected in our panel of PDC, which is a result largely concordant with previous studies on primary or xenografted PDC; b) the detection of sequence mutations of *DPC4* in PDC is at variance with a previous report on primary PDC and in agreement with the findings with xenografted PDC; c) alterations in K-*ras*, *p53*, *p16* and *DPC4* were found in a proportion of AVC and were virtually absent in PET, PAC, SPT or IPMT, the latter only showing relatively frequent K-*ras* mutations; d) the inactivation of *DPC4* gene by additional mechanisms such as homozygous deletion could be addressed indirectly by using immunohistochemistry, which showed the absence of the protein in about half of PDC and AVC and its expression in PAC, SPT, IPMT or PET. The extreme rarity of *p16* alterations and the lack of *DPC4* inactivation in PET are at variance with previous reports.

### Primary ductal cancers

As expected, in our series of 34 primary PDC we found mutations in K-*ras* and *p53* at a frequency largely concordant with those observed in previous studies on primary cancers (Almoguera et al, 1988; Hruban et al, 1993; Lemoine et al, 1992; Pellegata et al, 1994; Redston et al, 1994; Scarpa et al, 1993a, 1994a). Alterations of the *p16* gene were found in 13/34 (38%) cases, including 8 mutations (23%) and 5 cases with promoter methylation (15%). The *p16* mutational frequency of 23% in our cases is

© 2001 Cancer Research Campaign

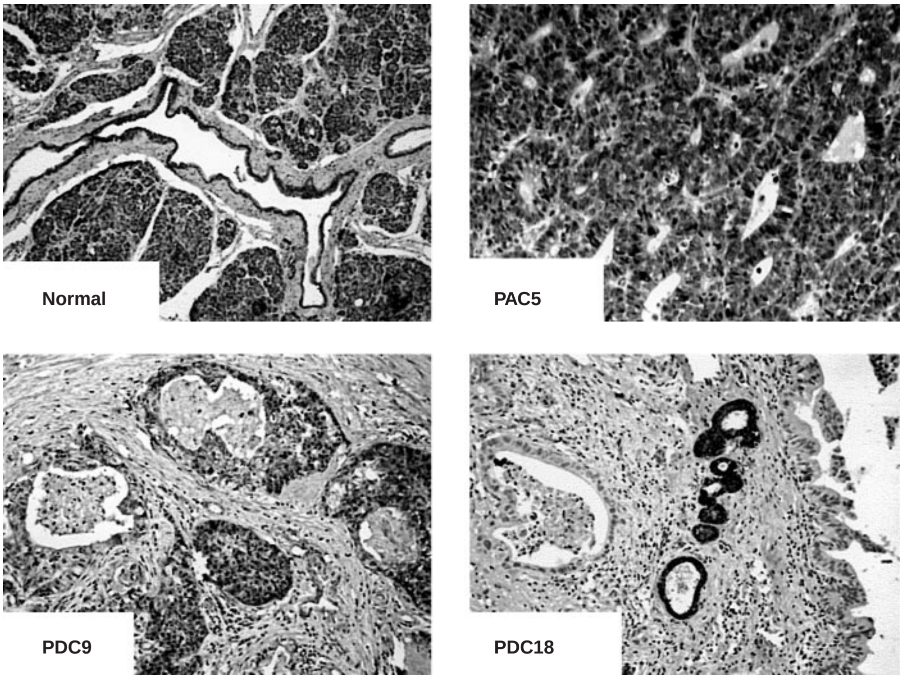

**Figure 5** Representative examples of immunohistochemistry with anti-DPC4 antibodies. Acinar and ductal cells of normal pancreas show positive staining, as do the shown cases of acinar cancer (PAC5) and pancreatic ductal carcinoma (PDC9). Pancreatic ductal cancer PDC18 is immunonegative in good agreement with the 17% mutational frequency found in an earlier study on 30 primary PDC (Huang et al, 1996) and reasonably close to the 35% found in 40 xenografts (Rozenblum et al, 1997). The present study is the first to address the frequency of *p16* promoter methylation in primary PDC and the results are in excellent agreement with the 15–18% observed in xenografted PDC and cell-lines (Schutte et al, 1997; Ueki et al, 2000). Mutations in *DPC4* were found in 9% of cases, at variance with the only other study using primary cancers where no mutations were found in 45 PDC (Bartsch et al, 1999a) and in agreement with the 13% mutations found in a total of 45 xenografts (Rozenblum et al, 1997; Villaneueva et al, 1998).

Homozygous deletions are frequent inactivating events of *p16* and *DPC4* that can be easily detected in xenografted cancers, cell lines or short term cultures (Caldas et al, 1994; Hahn et al, 1996; Huang et al, 1996; Villanueva et al, 1998; Jonson et al, 1999). Microdissected primary cancers are well suited for microsatellite analysis and enabled us to demonstrate unequivocal allelic losses on 9p, 17p and 18q in PDC in a high proportion of cases. However, it is particularly problematic to assay primary PDC for homozygous deletion, as their detection would require a cancer cellularity approaching 100%. This cannot be achieved in the large majority of PDC (compare panels A and B of Figure 4) due to the high admixture with nonneoplastic cells.

Immunohistochemistry however could be used as an alternative to reveal *p16* and *DPC4* inactivation due to homozygous deletion. Unfortunately, the status of *p16* could not be reliably assessed by this method. In fact, we have used 3 different antibodies with different antigen-retrieval methods and enhancing procedures, all with unsatisfactory results. It is also worth mentioning that in the report from Wilentz et al, no normal pancreatic cell or structure was immunostained, only a number of cells in hyperplastic ductal epithelia were positive and the authors themselves reported difficulty in interpretation due to significant cytoplasmic background staining (Wilentz et al, 1998). Similar problems with p16 immunohistochemistry using 4 different commercial antibodies has also been recently reported (Geradts et al, 2000). However, immunohistochemical staining for DPC4 consistently found positive staining in all normal pancreatic acinar, ductal and islet cells. Of particularly interest, 47% of PDC showed negative staining for the protein. DPC4 immunohistochemistry has been recently shown to correlate with inactivation of the gene in more than 90% of cases (Wilentz et al, 2000). This would provide additional evidence that homozygous deletion is a major mechanism of *DPC4* inactivation in ductal cancers.

### Intraductal papillary mucinous tumours

To date, K-*ras* mutations have been found in IPMT at varying frequency, ranging from 30 to 70% (Sessa et al, 1994; Satoh et al, 1996; Kondo et al, 1997; Z'Graggen et al, 1997). *p53* mutations are rare and associated with the invasive component of the tumour (Sessa et al, 1994). In 10 cryostat-enriched cases of IPMT, 7 of which had an invasive cancer component, we found 3 K-*ras* mutations and no alteration of *p16*, *p53* or *DPC4*. It would seem reasonable to consider IPMT a tumour entity with molecular targets involved in its pathogenesis distinct from those of ductal carcinoma. The low frequency of LOH found on chromosomal arms 9p, 17p, and 18q further substantiates this supposition. As expected from these studies, all cases also stained positively for DPC4.

### Acinar cancers

In PAC, mutations in K-*ras* are exceedingly rare (Hoorens et al, 1993; Terhune et al, 1994) and *p53* mutations have not been found in 3 cases previously reported (Pellegata et al, 1994). Our 6 PAC showed no K-*ras* nor *p16* mutations and frequent allelic losses on chromosomal arms 17p and 18q. However, our mutational analysis showed that the targets of these chromosomal losses are not *p53* nor *DPC4*, the latter result being additionally substantiated by immunohistochemistry.

*© 2001 Cancer Research Campaign*

**Table 1** Molecular alterations of K-*ras*, *p53*, *p16* and *DPC4* in primary pancreatic ductal cancers*

| | K-*ras* | | *p53* | | | *p16* | | | *DPC4* | | | |
|---|---|---|---|---|---|---|---|---|---|---|---|---|
| | Alteration | Predicted effect | Alteration | Predicted effect | Allelic Loss § | Alteration | Predicted effect | Allelic Loss § | Alteration | Predicted effect | Allelic Loss § | IHC** |
| PDC3 | c35G>A | G12D | c471–476del5 bp | frameshift | LOH | none | | NI | none | | NI | + |
| PDC4 | c35G>A | G12D | none | | ret | c269insT | frameshift | LOH | none | | LOH | + |
| PDC5 | none | | c746G>T | R249M | LOH | none | | ret | none | | ret | + |
| PDC6 | none | | c796G>T | G266X | NI | none | | ret | none | | LOH | + |
| PDC9 | none | | none | | ret | methylated | absent | ret | none | | ret | + |
| PDC10 | c35G>T | G12V | c718delA | frameshift | LOH | none | | ret | none | | LOH | + |
| PDC11 | c34G>A | G12S | none | | LOH | c128insA | Y44X | LOH | c1320del 58bp> TG | frameshift | LOH | – |
| PDC13 | c35G>T | G12V | none | | LOH | none | | ret | c1798C> T | polymorphism | LOH | – |
| PDC14 | c35G>T | G12V | c657delC | frameshift | LOH | none | | LOH | none | | LOH | + |
| PDC15 | c35G>A | G12D | none | | LOH | none | | LOH | none | | ret | + |
| PDC16 | c35G>T | G12V | c747G>T | R249S | LOH | c141C>A | P48T | LOH | none | | LOH | + |
| PDC17 | c35G>T | G12V | none | | ret | methylated | absent | ret | none | | LOH | not done |
| PDC18 | c35G>T | G12V | c817C>T | R273C | LOH | none | | LOH | c1798C>T | polymorphism | NI | – |
| PDC19 | c35G>T | G12V | c395A>G | K132R | LOH | none | | LOH | none | | LOH | – |
| PDC20 | c35G>A | G12D | c454–455delCC | frameshift | NI | none | | LOH | none | | LOH | – |
| PDC21 | c35G>A | G12D | c745A>G | R249G | ret | none | | LOH | none | | ret | not done |
| PDC22 | c35G>T | G12V | none | | ret | methylated | absent | LOH | none | | LOH | not done |
| PDC23 | c35G>A | G12D | none | | LOH | none | | ret | c1086T>C | polymorphism | ret | + |
| PDC24 | none | | none | | ret | c442G>A | polymorphism | ret | none | | ret | – |
| PDC25 | c35G>T | G12V | c729ins7bp | frameshift | LOH | none | | ret | none | | ret | – |
| PDC26 | c35G>T | G12V | none | | LOH | methylated | absent | LOH | none | | ret | not done |
| PDC27 | c35G>A | G12D | none | | LOH | none | | LOH | c1569C>G | CS23W | LOH | – |
| PDC28 | c35G>A | G12D | none | | NI | none | | ret | none | | ret | – |
| PDC29 | c35G>A | G12D | c530C>G | P177R | LOH | c171–177del | in-frame del | LOH | none | | LOH | – |
| PDC30 | c35G>A | G12D | none | | LOH | c230–231delCT | frameshift | LOH | none | | LOH | + |
| PDC31 | none | | none | | LOH | c328G>A | W110X | LOH | none | | LOH | + |
| PDC32 | none | | none | | LOH | none | | LOH | none | | LOH | + |
| PDC33 | c35G>T | G12V | c742G>A | R248Q | LOH | c284T>G | L94R | LOH | c1477G>A | D493N | LOH | – |
| PDC34 | c35G>T | G12V | c818G>A | R273H | LOH | none | | LOH | none | | NI | +/– |
| PDC35 | c34G>C | G12R | c818G>T | R273L | LOH | methylated | absent | LOH | none | | LOH | + |
| PDC36 | c34G>C | G12R | c844C>T | R282W | LOH | c165ins10 bp | frameshift | LOH | none | | ret | + |
| PDC37 | c35G>A | G12D | none | | LOH | none | | LOH | none | | LOH | – |
| PDC38 | c35G>A | G12D | c641A>G | H214R | LOH | c442G>A | polymorphism | ret | none | | ret | – |
| PDC39 | c35G>T | G12V | c524G>A | R175H | ret | none | | LOH | none | | LOH | + |
| | 28/34 82% | | 18/34 53% | | 24/31 77% | 13/34 38% | | 22/33 67% | 3/34 9% | | 20/31 65% | 14/30 47% |

*Mutations of K-*ras* and *p53* and allelic loss data on selected tumours are from Scarpa et al, 1993 and Achile et al, 1996. See Materials and methods for details. **IHC immunohistochemistry: +, positive staining neoplastic cells: –, negative staining neoplastic cells; +/– focal positive staining. § LOH, loss of heterozygosity: ret, retention of alleles; NI, noninformative.

### Solid pseudopapillary tumours

Neither *ras* gene family mutations nor alterations in *p53* have been found in SPT (Lee et al, 1997; Bartsch et al, 1998). Our 5 cases confirmed this data. We found no alterations in either *p16* or *DPC4* and no allelic losses on either 9p, 17p or 18q. Immunohistochemistry showed positive staining for DPC4, as expected. Thus, the molecular events leading to this peculiar entity remain elusive.

### Ampulla of Vater cancers

Our series of 16 AVC was composed of small neoplasms (<3 cm) of unequivocal ampullary origin, and cases showing microsatellite instability were excluded (Achille et al, 1997). Alterations were found in all 4 genes, with *p53* mutations being the most frequent event (60%), accompanied in the majority of cases by the loss of the second allele. K-*ras* mutations were detected in 47% of cases. One case had a mutation in the *DPC4* gene (6%) accompanied by the loss of the second allele, while 4 cases had inactivation of *p16* (25%) by promoter methylation. The most frequent allelic losses were on chromosomal arm 17p (63%), which has recently been found to be an independent prognostic factor among ampullary cancers at the same stage (Scarpa et al, 2000). Allelic loss on chromosomal arm 18q was a relatively frequent event (50%), while 9p LOH was infrequent (13%). Interestingly, 38% of cases showed negative staining for DPC4. This would imply that homozygous deletion of the *DPC4* gene is a frequent inactivating event in these tumours as for ductal cancers and reinforces the hypothesis that these two tumour types share common molecular pathways related to tumourigenesis and, possibly, progression of malignancy.

 *© 2001 Cancer Research Campaign*

**Table 2** Molecular alterations of K-*ras*, *p53*, *p16* and *DPC4* in primary pancreatic exocrine nonductal tumours*

| | K-*ras* | | *p53* | | | *p16* | | | *DPC4* | | | |
|---|---|---|---|---|---|---|---|---|---|---|---|---|
| IPMT** | Alteration | Predicted product | Alteration | Predicted product | Allelic Loss § | Alteration | Predicted product | Allelic Loss § | Alteration | Predicted product | Allelic Loss § | IHC*** |
| IPMT1** | none found | | none found | | ret | none found | | ret | none found | | ret | + |
| IPMT2** | none found | | none found | | ret | none found | | NI | none found | | ret | + |
| IPMT3** | none found | | none found | | ret | none found | | ret | none found | | ret | not done |
| IPMT4* | c35G>A | G12D | none found | | ret | none found | | ret | none found | | ret | not done |
| IPMT5*** | none found | | none found | | ret | none found | | ret | none found | | ret | + |
| IPMT6* | none found | | none found | | ret | none found | | ret | none found | | ret | + |
| IPMT7** | none found | | none found | | ret | none found | | ret | none found | | ret | + |
| IPMT8** | none found | | none found | | LOH | none found | | ret | none found | | ret | not done |
| IPMT9* | c35G>T | G12V | none found | | LOH | none found | | ret | none found | | LOH | + |
| IPMT10** | c35G>A | G12D | none found | | ret | none found | | ret | none found | | ret | – |
| | 3/10 | | 0/10 | | 2/10 | 0/10 | | 0/9 | 0/10 | | 1/10 | 0/7 |
| | 30% | | | | 20% | | | | | | 10% | |
| Acinar carcinoma | | | | | | | | | | | | |
| PAC1 | none found | | none found | | ret | none found | | ret | none found | | LOH | not done |
| PAC2 | none found | | none found | | LOH | none found | | LOH | none found | | LOH | + |
| PAC3 | none found | | none found | | NI | none found | | NI | none found | | ret | + |
| PAC4 | none found | | none found | | LOH | none found | | ret | none found | | ret | + |
| PAC5 | none found | | none found | | not done | none found | | not done | none found | | not done | + |
| PAC6 | none found | | none found | | ret | none found | | ret | none found | | ret | – |
| | 0/6 | | 0/6 | | 2/4 | 0/6 | | 1/4 | 0/6 | | 2/5 | 0/5 |
| | | | | | 50% | | | 25% | | | 40% | |
| Solid-pseudopapillary tumour | | | | | | | | | | | | |
| SPT1 | none found | | none found | | ret | none found | | ret | none found | | ret | + |
| SPT2 | none found | | none found | | ret | none found | | ret | none found | | ret | + |
| SPT3 | none found | | none found | | ret | none found | | ret | none found | | ret | + |
| SPT4 | none found | | none found | | not done | not done | | not done | not done | | not done | not done |
| SPT5 | none found | | none found | | ret | none found | | ret | none found | | ret | + |
| | 0/5 | | 0/5 | | 0/4 | 0/4 | | 0/4 | 0/4 | | 0/4 | 0/4 |

*Mutations of K-*ras* and *p53* on selected IPMT are from Sessa et al, 1994. See Materials and methods for details. **IPMT, Intraductal papillary-mucinous tumour: *, borderline; **, muconodular cancer component: *** ductal cancer component. ***IHC, immunohistochemistry; +, positive staining neoplastic cells; –, negative staining neoplastic cells; +/– focal positive staining. § LOH, loss of heterozygosity; ret, retention of alleles; NI, noninformative.

**Table 3** Molecular alterations of K-*ras*, *p53*, *p16* and *DPC4* in ampulla of Vater cancers*

| | K-ras | | p53 | | | p16 | | | DPC4 | | | |
|---|---|---|---|---|---|---|---|---|---|---|---|---|
| | Alteration | Predicted effect | Alteration | Predicted effect | Allelic Loss § | Alteration | Predicted effect | Allelic Loss § | Alteration | Predicted effect | Allelic Loss § | IHC** |
| AVC1 | none | | c733G>A | G245D | LOH | none | | ret | none | | LOH | + |
| AVC3 | none | | c659A>G | Y22OC | LOH | methylated | absent | ret | none | | LOH | – |
| AVC4 | c35G>A | G12D | none | | ret | none | | ret | none | | ret | + |
| AVC5 | none | | c733G>C | G245R | LOH | methylated | absent | ret | none | | ret | + |
| AVC6 | none | | c535C>T | H179Y | LOH | none | | ret | none | | ret | – |
| AVC7 | none | | none | | ret | none | | ret | none | | ret | + |
| AVC8 | none | | c657delC | frameshift | LOH | none | | LOH | none | | LOH | + |
| AVC9 | c35G>C | G12A | c517G>A | V173M | ret | none | | ret | none | | ret | + |
| AVC12 | none | | c845G>T | R282L | LOH | none | | ret | none | | LOH | – |
| AVC13 | c35G>A | G12D | c637C>T | R213X | LOH | c442G>A | polymorphism | ret | none | | LOH | – |
| AVC14 | c35G>A | G12D | none | | LOH | methylated | absent | ret | none | | LOH | – |
| AVC15 | none | | none | | LOH | c442G>A | polymorphism | ret | none | | ret | + |
| AVC17 | c35G>A | G12D | none | | ret | none | | LOH | none | | ret | + |
| AVC19 | c35G>A | G12D | c524G>A | R175H | ret | none | | ret | c1050-1065del | in-frame del | LOH | +/– |
| AVC60 | not done | | not done | | ret | none | | ret | none | | ret | + |
| AVC61 | c35G>C | G12A | none | | LOH | methylated | absent | ret | none | | LOH | + |
| | 7/15 | | 9/15 | | 10/16 | 4/16 | | 2/16 | 1/16 | | 8/16 | 6/16 |
| | 47% | | 60% | | 63% | 25% | | 13% | 6% | | 50% | 38% |

*Mutations of K-*ras* and *p53* and some allelic loss data on tumours AVC1-60 are from Scarpa et al, 2000. See Materials and methods for details. **IHC, immunohistochemistry; +, positive staining neoplastic cells; – , negative staining neoplastic cells; +/- focal positive staining. § LOH, loss of heterozygosity; ret, retention of alleles; NI, noninformative.

**Table 4** Molecular alterations of K-*ras*, *p53*, *p16* and *DPC4* in pancreatic endocrine tumours*

| | K-*ras* | | *p53* | | | *p16* | | | *DPC4* | | | |
|---|---|---|---|---|---|---|---|---|---|---|---|---|
| | **Alteration** | **Predicted product** | **Alteration** | **Predicted product** | **Allelic Loss §** | **Alteration** | **Predicted product** | **Allelic Loss §** | **Alteration** | **Predicted product** | **Allelic Loss §** | **IHC**** |
| NF1 | none | | none | | ret | none | | ret | none | | LOH | + |
| NF2 | none | | none | | LOH | none | | ret | none | | LOH | + |
| NF3 | none | | none | | ret | none | | ret | none | | ret | + |
| NF4 | none | | none | | LOH | none | | ret | none | | ret | + |
| NF5 | none | | none | | ret | none | | ret | none | | ret | + |
| NF6* | none | | none | | LOH | none | | ret | none | | ret | + |
| NF7* | none | | none | | LOH | c442G>A | polymorphism | ret | none | | LOH | + |
| NF8** | none | | none | | LOH | none | | ret | none | | ret | + |
| NF9** | none | | c709delG | frameshift | LOH | none | | LOH | none | | ret | + |
| NF10* | c35G>A | G12D | none | | LOH | none | | LOH | none | | LOH | + |
| NF17* | none | | none | | ret | none | | LOH | none | | ret | + |
| NF18* | none | | none | | ret | c442G>A | polymorphism | LOH | none | | ret | not done |
| NF19 | none | | none | | LOH | none | | NI | none | | ret | + |
| NF20* | none | | none | | ret | none | | LOH | none | | ret | + |
| NF21* | none | | none | | LOH | none | | LOH | none | | LOH | not done |
| NF22** | none | | none | | ret | none | | ret | none | | LOH | + |
| NF23 | none | | none | | ret | none | | ret | none | | LOH | + |
| NF24* | none | | none | | ret | none | | ret | none | | ret | + |
| NF25 | none | | none | | ret | none | | LOH | none | | LOH | + |
| NF26* | none | | none | | LOH | none | | NI | none | | ret | + |
| NF27 | none | | none | | LOH | none | | LOH | none | | ret | + |
| NF28 | none | | none | | LOH | none | | ret | none | | ret | + |
| NF29** | none | | none | | ret | none | | LOH | none | | ret | + |
| NF30 | none | | none | | ret | none | | ret | none | | ret | not done |
| NF31 | none | | none | | LOH | none | | ret | none | | ret | + |
| NF32 | none | | none | | LOH | none | | LOH | none | | ret | + |
| NF33 | none | | none | | LOH | none | | ret | none | | LOH | + |
| NF34 | none | | none | | ret | none | | LOH | none | | ret | + |
| NF35 | none | | none | | ret | none | | ret | none | | ret | + |
| NF36 | none | | none | | ret | c442G>A | polymorphism | ret | none | | ret | + |
| | 1/30 | | 1/30 | | 15/30 | 0/30 | | 11/28 | 0/30 | | 9/30 | 0/27 |
| | 3% | | 3% | | 50% | | | 39% | | | 30% | |
| F1 | none | | none | | ret | none | | NI | none | | LOH | + |
| F2 | none | | none | | ret | none | | LOH | none | | ret | + |
| F3 | none | | none | | ret | none | | ret | none | | LOH | + |
| F4 | none | | none | | ret | none | | ret | none | | ret | + |
| F5 | none | | none | | ret | none | | ret | none | | ret | + |
| F6 | none | | none | | ret | none | | ret | none | | LOH | + |
| F11 | none | | none | | ret | g152T>A | splicing error | ret | none | | ret | not done |
| F12 | none | | none | | ret | none | | ret | none | | ret | + |
| F13 | none | | none | | ret | c442G>A | polymorphism | NI | none | | ret | not done |
| F14^ | none | | none | | ret | none | | ret | none | | LOH | + |
| F15^^ | not done | | not done | | not done | none | | not done | none | | not done | + |
| | 0/10 | | 0/10 | | 0/10 | 1/11 | | 1/8 | 0/11 | | 4/10 | 0/9 |
| | | | | | | 9% | | 13% | | | 40% | |

*NF, nonfunctional; F, functional; ^ViPoma; ^^Gastrinoma. Mutations of K-*ras* and *p53* and some allelic loss data on cases NF1–10 and F1-7 are from Beghelli et al, 1998. See materials and methods for details. **IHC, immunohistochemistry; +, positive staining neoplastic cells; – , negative staining neoplastic cells; +/– focal positive staining. Malignant case (see Methods). Malignant case with liver metastasis. § LOH, loss of heterozygosity; ret, retention of alleles; NI, noninformative.

### Endocrine tumours

Our data confirm the extreme rarity of mutations in K-*ras* and *p53* in these tumours (Beghelli et al, 1998; Ebert et al, 1998; Lam and L.O, 1998). The rarity of *p53* mutations accompanied by LOH on chromosome 17p in 50% of nonfunctional cases, but in none of the functional PET, supports a previous suggestion indicating the presence of a tumour suppressor gene other than *p53* on chromosomal arm 17p involved in nonfunctional PET tumourigenesis (Beghelli et al, 1998). The virtual absence of *p16* and *DPC4* alterations in our 41 cases was somewhat unexpected given the recent reports of high frequency of inactivation of these genes in PET (Muscarella et al, 1998; Bartsch et al, 1999b). In our 41 PETs, only one insulinoma showed a *p16* alteration and no case showed alteration in the *DPC4* gene, in spite of the finding of a moderately frequent LOH on chromosomal arms 9p and 18q, found in 39% and 30% of nonfunctional and 13% and 40% of functional tumours, respectively.

In a study of 12 PETs, the *p16* gene has been reported to be altered in 92% of cases (7/8 gastrinomas and 4/4 nonfunctional) by either methylation (58%) or homozygous deletion (42%)

 *© 2001 Cancer Research Campaign*

(Muscarella et al, 1998). In our series, only one insulinoma had a mutation in *p16* and none of the 41 PETs showed *p16* methylation. The sensitivity of our methylation-specific PCR rules out the possibilities that tumour heterogeneity or neoplastic cellularity might have biased this result. It is possible that *p16* inactivation by promoter methylation might be restricted to functional gastrinomas, as all the reported PETs showing *p16* methylation were of this subtype (Muscarella et al, 1998). Moreover, neoplastic cellularity is not as problematic in PET with respect to PDC and virtually all cases can be enriched to nearly 100%. We observed no homozygous deletions of the *p16* gene under identical, standard amplification conditions previously used (Muscarella et al, 1998). Although it cannot be excluded that a small proportion of PETs have homozygous deletion of *p16*, it would appear to be a rare event. Finally, although the A148T polymorphism in the *p16* gene in was found in 10% of patients, which is well above the expected frequency (1.8%) (Aitken et al, 1999), this sequence variant has been reported to be functionally silent (Reymond and Brent 1995) and was not of statistically significant risk in families with melanoma (Aitken et al, 1999).

A recent study reported *DPC4* alterations in 5 of 9 malignant nonfunctional PET and in none of 16 functional PET, where it was suggested that alteration of this gene was correlated with malignancy (Bartsch et al, 1999b). Only one of these 5 alterations resulted from homozygous deletion, with the remaining 4 being mutations. We observed neither mutations nor homozygous deletions in the *DPC4* gene in our series of 11 functional and 30 nonfunctional enriched tumours. The latter included 18 malignant cases, 10 of which had liver metastases. Thus, our data suggest that the *DPC4* is not likely to play a central role in neuroendocrine tumourigenesis and the previously reported data may be biased by serendipity due to the small number of cases studied (Bartsch et al, 1999b). The conclusions reached from our molecular analysis of the *DPC4* gene in PET is further strengthened by results from immunohistochemistry in which all cases tested stained positively. Thus, it would appear unlikely that homozygous deletion of the *DPC4* gene is a frequent event in PET.

### CONCLUSIONS

By the molecular analysis of 112 pancreatic tumours of different types, it can be inferred that only carcinomas arising from the epithelium of pancreatic ducts and their terminal excretory structure (ampulla of Vater) have common molecular features. The genetic pathways implicated in ductal cancer are not involved in the pathogenesis of exocrine nonductal or endocrine tumours. These neoplasms must therefore have distinct molecular pathways involved in tumourigenesis. This likelihood seems highly plausible when considered together with the fact that each tumour type has distinct pathological and clinical features, including dramatic differences in patient survival.

### ACKNOWLEDGEMENTS

This study was supported by grants from the Associazione Italiana Ricerca Cancro (AIRC) to AS, Milan, Italy; Consorzio Studi Universitari di Verona, Italy; confinanced grant from Verona University and Ministero Università e Ricerca Scientifica e Tecnologica (MURST. COFIN MM06158571 9806151968-99-06195987-9906218982). Rome, Italy; Imperial Cancer Research Fund, the Mike Stone Cancer Research Fund and Medical Research Council, UK; Ministero Sanita (Ricerca Finalizzata cl. ligs. 229/93), Rome, Italy and European Community grant BIOMED 2 CA-Contract No. BMH4-CT98-3805. The authors thank George Elia (ICRF Histopathology Service, London UK) for technical assistance with part of immunohistochemistry.

### REFERENCES

Achille A, Biasi MO, Zamboni G, Bogina G, Magalini AR, Pederzoli P, Perucho M and Scarpa A (1996a) Chromosome 7q allelic losses in pancreatic carcinoma. *Cancer Res* **56**: 3808–3813

Achille A, Scupoli MT, Magalini AR, Zamboni G, Romanelli MG, Orlandini S, Biasi MO, Lemoine NR, Accolla RS and Scarpa A (1996b) APC gene mutations and allelic losses in sporadic ampullary tumours: evidence of genetic difference from tumours associated with familial adenomatous polyposis. *Int J Cancer* **68**: 305–312

Achille A, Biasi MO, Zamboni G, Bogina G, Iacono C, Talamini G, Capella G and Scarpa A (1997) Cancers of the papilla of Vater: mutator phenotype is associated with good prognosis. *Clin Cancer Res* **3**: 1841–1847

Achille A, Baron A, Zamboni G, Di Pace C, Orlandini S and Scarpa A (1998) Chromosome 5 allelic losses are early events in tumours of the papilla of Vater and occur at sites similar to those of gastric cancer. *Br J Cancer* **78**: 1653–1660

Aitken J, Welch J, Duffy D, Milligan A, Green A, Martin N and Hayward N (1999) CDKN2A variants in a population-based sample of Queensland families with melanoma. *J Natl Cancer Inst* **91**: 446–452

Almoguera C, Shibata D, Forrester K, Martin J, Arnheim N and Perucho M (1988) Most human carcinomas of the exocrine pancreas contain mutant c-K-ras genes. *Cell* **53**: 549–554

Barton CM, Staddon SL, Hughes CM, Hall PA, O'Sullivan C, Klöppel G, Theis B, Russell C, Neoptolemos J, Williamson RCN, Lane DP, Lemoine NR. (1991) Abnormalities of the p53 tumour suppressor gene in human pancreatic cancer. *Br J Cancer* **64**: 1076–1082

Bartsch D, Bastian D, Barth P, Schudy A, Nies C, Kisker O, Wagner HJ and Rothmund M (1998) K-ras oncogene mutations indicate malignancy in cystic tumors of the pancreas. *Ann Surg* **228**: 79–86

Bartsch D, Barth P, Bastian D, Ramaswamy A, Gerdes B, Chaloupka B, Deiss Y, Simon B and Schudy A (1999a) Higher frequency of DPC4/Smad4 alterations in pancreatic cancer cell lines than in primary pancreatic adenocarcinomas. *Cancer Lett* **139**: 43–49

Bartsch D, Hahn SA, Danichevski KD, Ramaswamy A, Bastian D, Galehdari H, Barth P, Schmiegel W, Simon B and Rothmund M (1996b) Mutations of the DPC4/Smad4 gene in neuroendocrine pancreatic tumors. *Oncogene* **18**: 2367–2371

Beghelli S, Pelosi G, Zamboni G, Falconi M, Iacono C, Bordi C and Scarpa A (1998) Pancreatic endocrine tumours: evidence for a tumour suppressor pathogenesis and for a tumour suppressor gene on chromosome 17p. *J Pathol* **186**: 41–50

Caldas C, Hahn SA, Costa LTd, Redston MS, Schutte M, Seymour AB, Weinstein CL, Hruban RH, Yeo CJ and Kern SE (1994) Frequent somatic mutations and homozygous deletions of the p16 (MTS1) gene in pancreatic adenocarcinoma. *Nature Genet* **8**: 27–32

Ebert MP, Hoffmann J, Schneider-Stock R, Kasper HU, Schulz HU, Lippert H, Roessner A and Malfertheiner P (1998) Analysis of K-ras gene mutations in rare pancreatic and ampullary tumours. *Eur J Gastroenterol Hepatol* **10**: 1025–1029

Geradts J, Hruban RH, Schutte M, Kern SE and Maynard R (2000) Immunohistochemical p161NK4a analysis of archival tumors with deletion, hypermethylation, or mutation of the CDKN2/MTS1 gene. A comparison of four commercial antibodies. *Appl Immunohistochem Molecul Morphol* **8**: 71–79

Hahn SA, Schutte M, Hoque AT, Moskaluk CA, da Costa LT, Rozenblum E, Weinstein CL, Fischer A, Yeo CJ, Hruban RH and Kern SE (1996) DPC4, a candidate tumor suppressor gene at human chromosome 18q21.1. *Science* **271**: 350–353

Hahn SA, Bartsch D, Schroers A, Galehdari H, Becker M, Ramaswamy A, Schwarte-Waldhoff I, Maschek H and Schmiegel W (1998) Mutations of the DPC4/Smad4 gene in biliary tract carcinoma. *Cancer Res* **58**: 1124–1126

Herman JG, Graff JR, Myohanen S, Nelkin BD and Baylin SB (1996) Methylation-specific PCR: a novel PCR assay for methylation status of CpG islands. *Proc Natl Acad Sci USA* **93**: 9821–9826

Hoorens A, Lemoine NR, McLellan E, Morohoshi T, Kamisawa T, Heitz PU, Stamm B, Ruschoff J, Wiedenmann B and Kloppel G (1993) Pancreatic acinar cell carcinoma. An analysis of cell lineage markers, p53 expression, and Ki-ras mutation. *Am J Pathol* **143**: 685–698

Hruban RH, van Mansfeld AD, Offerhaus GJ, van Weering DH, Allison DC, Goodman SN, Kensler TW, Bose KK, Cameron JL and Bos, JL (1993) K-ras oncogene activation in adenocarcinoma of the human pancreas. A study of 82 carcinomas using a combination of mutant-enriched polymerase chain reaction analysis and allele-specific oligonucleotide hybridization. *Am J Pathol* **143**: 545–554

Huang L, Goodrow TL, Zhang SY, Klein-Szanto AJ, Chang H and Ruggeri BA (1996) Deletion and mutation analyses of the P16/MTS-1 tumor suppressor gene in human ductal pancreatic cancer reveals a higher frequency of abnormalities in tumor-derived cell lines than in primary ductal adenocarcinomas. *Cancer Res* **56**: 1137–1141

Jonson T, Gorunova L, Dawiskiba S, Andren-Sandberg A, Stenman G, ten Dijke P, Johansson B and Hoglund M (1999) Molecular analyses of the 15q and 18q SMAD genes in pancreatic cancer. *Genes Chromosomes Cancer* **24**: 62–71

Kalthoff H, Schmiegel W, Roeder C, Kasche D, Schmidt A, Lauer G, Thiele HG, Honold G, Pantel K, Riethmuller G and et al. (1993) p53 and K-RAS alterations in pancreatic epithelial cell lesions. *Oncogene* **8**: 289–298

Klimstra DS, Heffess CS, Oertel JE and Rosai J (1992) Acinar cell carcinoma of the pancreas. A clinicopathologic study of 28 cases. *Am J Surg Pathol* **16**: 815–837

Klöppel G, Solcia E, Longnecker D, Capella C and Sobin L (1996) *Histological typing of tumours of the exocrine pancreas*. International Histological Classification of Tumours. Springer-Verlag: Berlin

Kondo H, Sugano K, Fukayama N, Hosokawa K, Ohkura H, Ohtsu A, Mukai K and Yoshida S (1997) Detection of K-ras gene mutations at codon 12 in the pancreatic juice of patients with intraductal papillary mucinous tumors of the pancreas. *Cancer* **79**: 900–905

Lam KY and Lo CY (1998) Role of p53 tumor suppressor gene in pancreatic endocrine tumors of Chinese patients. *Am J Gastroenterol* **93**: 1232–1235

Lee WY, Tzeng CC, Chen RM, Tsao CJ, Tseng JY and Jin YT (1997) Papillary cystic tumors of the pancreas: assessment of malignant potential by analysis of progesterone receptor, flow cytometry, and ras oncogene mutation. *Anticancer Res* **17**: 2587–2591

Lemoine NR, Jain S, Hughes CM, Staddon SL, Maillet B, Hall PA and Kloppel G (1992) Ki-ras oncogene activation in preinvasive pancreatic cancer. *Gastroenterology* **102**: 230–236

Moore PS, Rigaud G, Baron A and Scarpa A (2000a) Two novel polymorphisms, c1086T>C and c1798C>T, in the MADH4/DPC4 gene. *Hum Mutat (Online)* **15**: 485–486

Moore PS, Zamboni G, Falconi M, Bassi C and Scarpa A (2000b) A novel germline mutation, P48T, in the CDKN2A/p16 gene in a patient with pancreatic carcinoma. *Hum Mutat (Online)*, in press

Muscarella P, Melvin WS, Fisher WE, Foor J, Ellison EC, Herman JG, Schirmer WJ, Hitchcock CL, DeYoung BR and Weghorst CM (1998) Genetic alterations in gastrinomas and nonfunctioning pancreatic neuroendocrine tumors: an analysis of p16/MTS1 tumor suppressor gene inactivation. *Cancer Res* **58**: 237–240

Naumann M, Savitskaia N, Eilert C, Schramm A, Kalthoff H and Schmiegel W (1996) Frequent codeletion of p16/MTS1 and p15/MTS2 and genetic alterations in p16/MTS1 in pancreatic tumors. *Gastroenterology* **110**: 1215–1224

Nielsen GP, Stemmer-Rachamimov AO, Shaw J, Roy JE, Koh J and Louis DN (1999) Immunohistochemical survey of p16INK4A expression in normal human adult and infant tissues. *Lab Invest* **79**: 1137–1143

Pellegata NS, Sessa F, Renault B, Bonato M, Leone BE, Solcia E and Ranzani GN (1994) K-ras and p53 gene mutations in pancreatic cancer: ductal and nonductal tumors progress through different genetic lesions. *Cancer Res* **54**: 1556–1560

Redston MS, Caldas C, Seymour AB, Hruban RH, da Costa L, Yeo CJ and Kern SE (1994) p53 mutations in pancreatic carcinoma and evidence of common involvement of homocopolymer tracts in DNA microdeletions. *Cancer Res* **54**: 3025–3033

Reymond A and Brent R (1995) p16 proteins from melanoma-prone families are deficient in binding to Cdk4. *Oncogene* **11**: 1173–1178

Rozenblum E, Schutte M, Goggins M, Hahn SA, Panzer S, Zahurak M, Goodman SN, Sohn TA, Hruban RH, Yeo CJ and Kern SE (1997) Tumor-suppressive pathways in pancreatic carcinoma. *Cancer Res* **57**: 1731–1734

Satoh K, Shimosegawa T, Moriizumi S, Koizumi M and Toyota T (1996) K-ras mutation and p53 protein accumulation in intraductal mucin-hypersecreting neoplasms of the pancreas. *Pancreas* **12**: 362–368

Scarpa A, Capelli P, Mukai K, Zamboni G, Oda T, Iacono C and Hirohashi S (1993a) Pancreatic adenocarcinomas frequently show p53 gene mutations. *Am J Pathol* **142**: 1534–1543

Scarpa A, Capelli P, Zamboni G, Oda T, Mukai K, Bonetti F, Martignoni G, Iacono C, Serio G and Hirohashi S (1993b) Neoplasia of the ampulla of Vater, Ki-ras and p53 mutations. *Am J Pathol* **142**: 1163–1172

Scarpa A, Capelli P, Villaneuva A, Zamboni G, Lluis F, Accolla R, Mariuzzi G and Capella G (1994a) Pancreatic cancer in Europe: Ki-ras gene mutation pattern shows geographical differences. *Int J Cancer* **57**: 167–171

Scarpa A, Zamboni G, Achille A, Capelli P, Bogina G, Iacono C, Serio G and Accolla RS (1994b) ras-family gene mutations in neoplasia of the ampulla of Vater. *Int J Cancer* **59**: 39–42

Scarpa A, Di Pace C, Talamini G, Iacono C, Falconi M, Achille A, Baron A and Zamboni G (2000) Chromosome 17p loss and prognosis of cancer of papilla of Vater. *Gut* **46**: 842–848

Schutte M, Hruban RH, Geradts J, Maynard R, Hilgers W, Rabindran SK, Moskaluk CA, Hahn SA, Schwarte-Waldhoff I, Schmiegel W, Baylin SB, Kern SE and Herman JG (1997) Abrogation of the Rb/p16 tumor-suppressive pathway in virtually all pancreatic carcinomas. *Cancer Res* **57**: 3126–3130

Sessa F, Solcia E, Capella C, Bonato M, Scarpa A, Zamboni G, Pellegata N, Ranzani G, Rickaert F and Klöppel G (1994) Intraductal papillary-mucinous pancreatic tumors are phenotypically, genetically and behaviorally distinct growths. An analysis of tumor cell phenotype, K-ras and p53 gene mutations. *Virchows Archiv* **425**: 357–367

Solcia E, Capella C and Klöppel G (1997) *Tumors of the pancreas*. Vol. 20. Atlas of tumor pathology. Armed Forces Institute of Pathology: Washington, DC

Sorio C, Baron A, Orlandini S, Zamboni G, Pederzoli P, Huebner K and Scarpa A (1999) The FHIT gene is expressed in pancreatic ductular cells and is altered in pancreatic cancers. *Cancer Res* **59**: 1308–1314

Terhune PG, Heffess CS and Longnecker DS (1994) Only wild-type c-Ki-ras codons 12, 13, and 61 in human pancreatic acinar cell carcinomas. *Mol Carcinog* **10**: 110–114

Ueki T, Toyota M, Sohn T, Yeo CJ, Issa JP, Hruban RH and Goggins M (2000). Hypermethylation of multiple genes in pancreatic adenocarcinoma. *Cancer Res*, **60**: 1835–9

Villanueva A, Garcia C, Paules AB, Vicente M, Megias M, Reyes G, de Villalonga P, Agell N, Lluis F, Bachs O and Capella G (1998) Disruption of the antiproliferative TGF-beta signaling pathways in human pancreatic cancer cells. *Oncogene* **17**: 1969–1978

Wilentz RE, Geradts J, Maynard R, Offerhaus GJ, Kang M, Goggins M, Yeo CJ, Kern SE and Hruban RH (1998) Inactivation of the p16 (INK4A) tumor-suppressor gene in pancreatic duct lesions: loss of intranuclear expression. *Cancer Res* **58**: 4740–4744

Wilentz RE, Su GH, Dai JL, Sparks AB, Argani P, Sohn TA, Yeo CJ, Kern SE and Hruban RH (2000) Immunohistochemical labeling for dpc4 mirrors genetic status in pancreatic adenocarcinomas: a new marker of DPC4 inactivation. *Am J Pathol* **156**: 37–43

Zamboni G, Bonetti F, Scarpa A, Pelosi G, Doglioni C, Iannucci A, Castelli P, Balercia G, Aldovini D, Bellomi A, Iacono C, Serio G and Mariuzzi G (1993) Expression of progesterone receptors in solid-cystic tumour of the pancreas: a clinicopathological and immunohistochemical study of ten cases. *Virchows Archiv A* **423**: 425–431

Z'Graggen K, Rivera JA, Compton CC, Pins M, Werner J, Fernandez-del Castillo C, Rattner DW, Lewandrowski KB, Rustgi AK and Warshaw AL (1997) Prevalence of activating K-ras mutations in the evolutionary stages of neoplasia in intraductal papillary mucinous tumors of the pancreas. *Ann Surg* **226**: 491–498

Zhang SY, Klein-Szanto AJ, Sauter ER, Shafarenko M, Mitsunaga S, Nobori T, Carson DA, Ridge JA and Goodrow TL (1994) Higher frequency of alterations in the p16/CDKN2 gene in squamous cell carcinoma cell lines than in primary tumors of the head and neck. *Cancer Res* **54**: 5050–5053

 *© 2001 Cancer Research Campaign*
